# Risk Factors for Residual Vitreous Cortex at the Fovea after Posterior Vitreous Detachment during Vitrectomy in Ocular Trauma

**DOI:** 10.1155/2019/4312958

**Published:** 2019-07-28

**Authors:** Anan Wang, Zhenquan Zhao, Qihua Xu, Yaohua Wang, Hongfei Liao

**Affiliations:** ^1^Affiliated Eye Hospital of Nanchang University, Nanchang, Jiangxi Province, China; ^2^Eye Hospital of Wenzhou Medical College, Wenzhou, Zhejiang Province, China

## Abstract

**Purpose:**

To investigate the frequency and patterns of the residual vitreous cortex (RVC) at the fovea after posterior vitreous detachment (PVD) during vitrectomy after ocular trauma and the risk factors associated with its occurrence.

**Methods:**

A cross-sectional, multicenter, and observational study was conducted in 72 consecutive patients who underwent pars plana vitrectomy after ocular trauma. In patients with PVD after vitrectomy, RVC patterns were visualized using triamcinolone acetonide staining and were classified as diffuse or focal. A multivariate logistic regression analysis was performed to evaluate the association between RVC and various factors, such as the patient's age, preoperative visual acuity, days after injury, the existence of spontaneous PVD, and the type of injury prior to vitrectomy.

**Results:**

Of the 72 eyes with trauma, 35 eyes (48.6%) showed RVC on the macula, of which 19 (54.3%) were of the diffuse type and 16 (45.7%) were of the focal type. A multivariate logistic regression analysis showed that age (OR = 0.933, *P*=0.025) and days after injury (OR = 0.862, *P*=0.013) had a significant impact on RVC.

**Conclusions:**

RVC at the fovea is very common after spontaneous or surgical PVD during vitrectomy. Younger patients are more likely to show RVC if vitrectomy is performed soon after injury.

## 1. Introduction

The posterior vitreous cortex is now recognized to be a major component of epiretinal membranes [1]. Therefore, a critical step in successful vitrectomy is the complete removal of the vitreous cortex from the retina in order to reduce postoperative complications [2]. Posterior vitreous detachment (PVD) is thought to play a role in pars plana vitrectomy. Residual vitreous cortex (RVC) after PVD can provide a scaffold for cell proliferation and produce traction, which can lead to vitreoretinal interface disorders that influence final visual acuity [3, 4].

Intravitreous triamcinolone acetonide (TA) staining has been widely used to visualize the transparent vitreous cortex and facilitates complete separation of the posterior vitreous cortex from the retina. However, even after spontaneous or surgical PVD in pars plana vitrectomy surgery, some residual cortex may be observed. This phenomenon has not been reported in ocular trauma, and risk factors for RVC are not yet known.

The purpose of this study was to determine the incidence of RVC after spontaneous or surgical PVD in patients who had undergone vitrectomy and to analyze the factors that influence the risk for RVC occurrence.

## 2. Methods

### 2.1. Patients

This is a cross-sectional and observational study of eyes from 72 consecutive patients who were diagnosed with ocular trauma and who underwent vitrectomy at the Eye Hospital of Wenzhou Medical College and the Affiliated Eye Hospital of Nanchang University between December 2013 and December 2015. Our study was approved by the Institutional Review Board of the Affiliated Eye Hospital of Nanchang University, designated by the Ministry of Health of China, and followed the ethical standards of the Declaration of Helsinki. The possible merits and risks of the present treatment were explained to the patients before surgery, and informed consent was obtained from all patients. The surgeon for each patient was determined randomly.

We excluded patients with (a) systemic diseases, such as diabetes and hypertension, (b) any previous retinal pathology and accepted vitrectomy surgery, (c) endophthalmitis, (d) proliferative vitreoretinopathy, (e) traumatic macular diseases, (f) injuries that had occurred more than 1 month earlier, (g) unsuccessful surgical PVD, and (h) any kind of intervention in the vitreous cavity like anti-VEGF injections during operation.

In all cases, an ophthalmic B-scan ultrasound was performed before vitrectomy. The patients were examined in a supine position on the bed with an ultrasound probe placed on the closed eyelid surface, with the minimum pressure. If necessary, gentle eye movements were maintained during the examination. In cases with vitreous opacities and obscuration, we judged whether a spontaneous PVD was present or not by ultrasound before the vitrectomy surgery. As described by McNicholas et al. and Almendárez et al. [5, 6], PVD in cases with ocular trauma was seen as a thin, undulating, mobile line that moved away from the posterior aspect of the globe during eye movements.

Patient data, such as age, sex, preoperative visual acuity, days after injury, post-injury operations, the existence of spontaneous PVD before vitrectomy, and the type of injury, which included closed globe injury and open globe injury, were collected. Preoperative visual acuity values were transformed to the logMAR scale. In cases of profound low vision or near blindness, as determined by perception of counting fingers, hand movements, light perception (LP), and no LP, visual acuity values were substituted by logMAR values of 1.7, 2.0, 2.5, and 3.0, respectively, as reported by Heimann et al. [7]. Days after injury were defined as the number of days between the injury and vitrectomy surgery. Post-injury operations were defined as the number of operations between the injury and vitrectomy surgery.

### 2.2. Surgical Procedure

**Figure 1:**
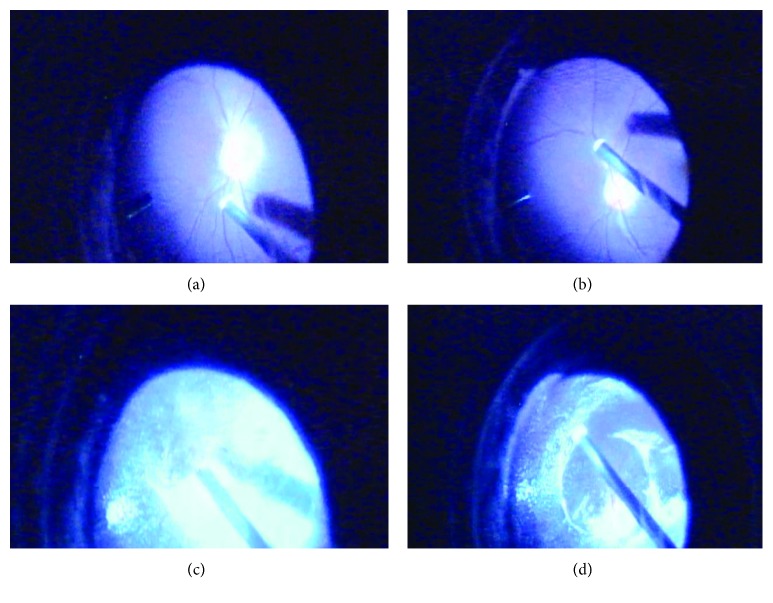
Formation of surgical posterior vitreous detachment (PVD). (a) The attraction of the vitreous cortex with high vacuum levels transmitted through the vitrectomy probe, over the optic nerve. (b) The vitreous cortex is gently pulled up by the vitrectomy probe. (c) Suspension of triamcinolone acetonide granules in the vitreous cavity. (d) Successful surgical PVD is confirmed by the appearance of a peripapillary Weiss ring.

All patients underwent a standard 3-port 23-gauge (G) pars plana vitrectomy. If there was no spontaneous PVD, a surgical PVD was created with high vacuum levels transmitted through the vitrectomy probe or flute needle, down from the vitreous cortex to over the optic nerve, and was confirmed by the appearance of a peripapillary Weiss ring (Figure 1). After the vitreous body was removed, maximum removal of the basal vitreous gel was achieved by pressing on the peripheral sclera. Next, 4 mg/0.1 ml suspended triamcinolone acetonide (TA, 40 mg/ml; Kunming Jida Pharmaceutical Co. Ltd.) was injected into the mid-vitreous cavity. After the TA granules adhered to the RVC, we washed out the excess granules in the vitreous cavity to visualize the RVC at the fovea. The RVC was identified and removed by flute needles or forceps. Finally, depending on the conditions, a fluid-air exchange, endolaser photocoagulation, or cryosurgery for retinal tears and degenerated areas, and tamponade with silicone oil, sulfur hexafluoride, or perfluoropropane gas were performed.

### 2.3. RVC Pattern

**Figure 2:**
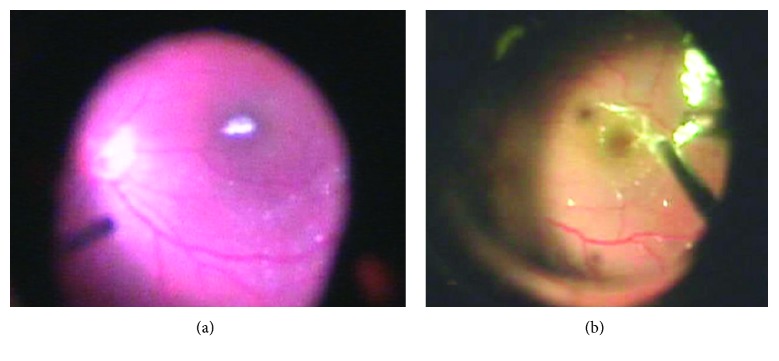
Patterns of the residual vitreous cortex (RVC): (a) focal type; (b) diffuse type.

After surgery, two retinal specialists watched the video of the surgery and confirmed the shape and the area of the RVC. Based on the area, the RVC was divided into the following two types: focal, with an RVC area smaller than 1 disc diameter, and diffuse, with an RVC area equal to or larger than 1 disc diameter (Figure 2).

### 2.4. Statistical Analysis

Continuous variables were expressed as mean ± standard deviation. We used the chi-squared test and Kruskal–Wallis test to compare clinical results in the absence and presence of RVC groups. The presence of RVC (no RVC = 0 and RVC = 1) is a dependent variable; multivariate logistic regression analysis was used to evaluate the effects of variables which were significant in the chi-squared test or Kruskal–Wallis test. The data were analyzed using the SPSS 19.0 software. Results with *P* < 0.05 were considered statistically significant.

## 3. Results

The study evaluated 72 consecutive cases who underwent pars plana vitrectomy after ocular trauma, of whom 63 (87.5%) were men and 9 (12.5%) were women. Of the eyes studied, 35 (48.6%) were right eyes and 37 (51.4%) were left eyes.

RVC was observed on the macula after spontaneous or surgical PVD during TA-assisted vitrectomy in 35 (48.6%) eyes. TA staining demonstrated that the RVC patterns were of the diffuse type in 19 (54.3%) eyes and of the focal type in 16 (45.7%) eyes.

Based on the presence or absence of RVC, we divided all cases into two groups.  Table 1 shows clinical findings of this study. Sex and operative times after injury were similar in the absence and presence of RVC groups (*P*=0.909 and *P*=0.452). However, the group with RVC comprised younger patients with better preoperative visual acuity, fewer days after injury, fewer cases of spontaneous PVD before vitrectomy, and a larger rate of open globe injury significantly (*P*=0.007, *P*=0.042, *P*=0.002, *P*=0.024, and *P*=0.004, respectively).

Table 2 shows the results of a multivariate logistic regression analysis. Age (odds ratio (OR): 0.933, 95% CI: 0.878–0.991, *P*=0.025) and days after injury (odds ratio (OR): 0.862, 95% CI: 0.767–0.969, *P*=0.013) were independent factors contributing to the occurrence of RVC, while preoperative visual acuity, spontaneous PVD, and injury type were found to have no significant effects on RVC.

## 4. Discussion

In this study, we have investigated the frequency and patterns of RVC at the fovea after PVD during vitrectomy for ocular trauma and the risk factors associated therewith.

The phenomenon of an RVC adhering to the retina after PVD is commonly observed with TA staining of the vitreous cortex [8]. Histopathological examination found that 44% of the eyes had an RVC at the fovea after spontaneous PVD [9]. Kimura et al. have reported a study of 9 patients with a rhegmatogenous retinal detachment (RRD) who had a premacular vitreous cortex with surgical PVD [10]. The percentage of diffuse and focal types of RVC was each 50%. In a study of patients with proliferative diabetic retinopathy (PDR), diabetic macular edema, branch retinal vein occlusion, and RRD showed that 40–90% of the cases had RVC, with the highest incidence among patients with PDR. While 42.9–88.9% of the cases had the diffuse type of RVC, 11.1–57.1% had the focal type [2]. Moreover, in 33 cases of RRD with spontaneous PVD, Chen et al. found that RVC staining was most frequently seen on the macula (10 of 23 cases, 43.5%) [11]. In another study, Cho et al. found that, of the 60 cases (75%) that showed RVC on the macula, 42 (70%) were of the diffuse type [12]. In this study, we observed that 48.6% of the cases showed macular RVC, of which 54.3% were of the diffuse type and 45.7% were of the focal type.

We propose that the occurrence of RVC at the fovea may be due to the following: First, the staining may be related to vitreoschisis [12]. An inaccurate perception of the posterior hyaloid membrane and transparent vitreous cortex can result in misidentification of the inner wall of the vitreoschisis cavity as the posterior vitreous wall. Second, synchysis is maculocentric in individuals with age-related PVD. The RVC layer at the fovea becomes extremely thin before a tear in the vitreous cortex triggers PVD. The thinness of this cortical layer and the tearing forces that initiate PVD are likely to cause the vitreous gel to attach to the macula [9, 13]. Finally, firm vitreoretinal adhesion to the macula is likely to leave remnants on the retinal surface [10], which has been confirmed in many clinical studies, in the form of macular holes after PVD and vitreoretinal traction syndrome [14, 15].

Sonoda et al. found that the diffuse type (88.9%) of RVC was more common compared to the focal type (11.1%) in diabetic patients after PVD for vitreopathy [2]. In nondiabetic patients, diffuse RVC was reported at a frequency of 42.9–70% [2, 10–12], while its incidence in our study was 54.3%. However, the mechanisms underlying the patterns of RVC in nondiabetic patients are unclear. Our finding that the incidence of the diffuse type is higher than that of the focal type of premacular RVC may be associated with the firm nature of the vitreoretinal adhesion. The mean patient age in this study was 37.71 years, an age at which vitreous liquefaction is inadequate and vitreoretinal adherence to the macula is tough, so that the majority of PVD has to be surgically induced. In younger individuals, the adhesion between the posterior hyaloid membrane and the internal limiting membrane is more extensive and stronger than the intravitreous adhesion [16]. These factors could explain the higher incidence of the diffuse type of RVC in our study.

It has been confirmed that eyes with RVC have a higher rate of epiretinal membranes after vitrectomy compared to eyes without RVC [12]. Therefore, the recognition of complete PVD and the factors affecting RVC are clinically relevant [1, 17]. Nevertheless, research into the factors that impact RVC after ocular trauma has been inadequate.

To address this, we used multivariate logistic regression analysis to evaluate the effects of age, preoperative visual acuity, days after injury, the existence of spontaneous PVD before vitrectomy, and the type of injury on the occurrence of RVC in traumatic eyes. Age and days after injury had a significant influence on the occurrence of RVC. Although patients with RVC on the macula were less common among cases with spontaneous PVD, those that had worse preoperative visual acuity, and those with closed globe injury, no statistically significant association was detected between preoperative visual acuity, the existence of spontaneous PVD, and injury type.

There are three known mechanisms by which age protects against RVC. First, age is closely related to vitreous liquefaction because age-related free radicals cause hyaluronan depolymerization, which leads to the destruction of the gel structure [18]. Next, the crosslinking of collagen fibers induced by free radicals can affect vitreous shrinkage [19, 20]. Last, with increasing age, vitreoretinal adhesion is weakened [16, 21]. Synchysis, vitreous shrinkage, and vitreoretinal adhesion are the main factors related to PVD and are associated with RVC.

In addition, the increasing number of days between the injury and vitrectomy showed a significant protective effect against RVC, which may be because of vitreous changes. After an eye injury, the intraocular structures change, and the blood-retinal barrier is broken. The blood components and cell mediation lead to vitreous shrinkage, causing PVD [22–24]. Therefore, with a greater delay in performing vitrectomy after the injury, fewer vitreous remnants remain.

The major limitations of this study include (a) a small sample size and (b) judgement of spontaneous PVD and RVC without objective standards. However, this study revealed that RVC frequently remains on the macula after PVD. The impact of various factors on the occurrence of RVC in traumatic eyes has also not been described previously. We believe these findings may be valuable for surgeons performing vitrectomy and can facilitate understanding of the pathological mechanism underlying vitreoretinopathy, allowing for a better clinical prognosis.

In conclusion, in ocular trauma, the presence of premacular RVC is very common, even after spontaneous or surgical PVD during vitrectomy. We show that older age and more days after injury are significant protective factors for RVC at the fovea. Further objective studies are needed to evaluate the difference in these influential factors, using larger sample sizes.

## Figures and Tables

**Table 1:** Clinical characteristics of ocular trauma patients with absence and presence of RVC.

	Absence of RVC	Presence of RVC		*P*
Number of eyes	37 (51.4%)	35 (48.6%)		
		Diffuse type	Focal type	
		19 (54.3%)	16 (45.7%)	
Age (years)	46.05 (±10.14)	37.71 (±10.67)		0.007
Sex (M/F)	32/5	31/4		0.909^*∗*^
Preoperative visual acuity (logMAR)	1.81 (±0.58)	1.57 (±0.62)		0.042
Days after injury	12.16 (±8.16)	6.66 (±4.33)		0.002
Operative times after injury	0.43 (±0.56)	0.49 (±0.51)		0.452^*∗*^
Spontaneous PVD	11 (29.7%)	3 (8.6%)		0.024
Injury type				0.004
Closed globe	14 (37.8%)	3 (8.6%)		
Open globe	23 (62.2%)	32 (91.4%)		

RVC = residual vitreous cortex; PVD = posterior vitreous detachment. Data displayed are either mean values with SDs for continuous variables or number and percentage for categorical variables in the absence and presence of RVC groups. Unmarked *P* values are detected by the Kruskal–Wallis test, while those marked with ^*∗*^ by the chi-squared test.

**Table 2:** Multivariate logistic regression analysis of factors independently contributing to presence of RVC.

Variables		OR (95% CI)	*P*
Dependent	Independent		
Presence of RVC	Age (years)	0.933 (0.878–0.991)	0.025
	Preoperative visual acuity (logMAR)	0.341 (0.104–1.120)	0.076
	Days after injury	0.862 (0.767–0.969)	0.013
	Spontaneous PVD	0.742 (0.142–3.873)	0.723
	Injury type	4.288 (0.942–19.519)	0.060

RVC = residual vitreous cortex; PVD = posterior vitreous detachment; OR = odds ratio.

## Data Availability

The datasets obtained and/or analyzed during the current study are available from the corresponding author on reasonable request (email: liaohongfei6000@163.com).
